# The Effect of Reduced Nitrogen Fertilizer Application on *japonica* Rice Based on Volatile Metabolomics Analysis

**DOI:** 10.3390/foods13203310

**Published:** 2024-10-18

**Authors:** Jiahao Wu, Qian Wang, Dong Zhang, Xiaoliang Duan, Hui Sun

**Affiliations:** 1 School of Health Science and Engineering, University of Shanghai for Science and Technology, Shanghai 200093, China; 15712806480@163.com; 2 Academy of National Food and Strategic Reserves Administration, Beijing 100037, China; wangq@ags.ac.cn (Q.W.); zd@ags.ac.cn (D.Z.); dxl@ags.ac.cn (X.D.)

**Keywords:** rice, nitrogen, reduction, volatile metabolomic, OPLS-DA, quality

## Abstract

Nitrogen is critical for rice yield and quality, but its overuse can be detrimental to efficiency and the environment. To identify changes in the quality of rice in response to the reduced application of nitrogen fertilizer, we carried out a comprehensive metabolomics study of SuiJing 18 using volatile metabolomics methods. Our results showed that SuiJing 18 had a total of 358 volatile metabolites, mainly lipids (16.25%), terpenoids (15.41%), heterocyclic compounds (15.13%), and hydrocarbons (13.45%). SuiJing 18 underwent significant changes in response to the reduced application of nitrogen fertilizer. Key sweet volatile compounds such as 4-methyl-benzeneacetaldehyde, hexyl acetate, and 2-methylnaphthalene were present at significantly higher levels when nitrogen fertilizer was applied at a rate of 68 kg of pure nitrogen per hectare, and their flavor characteristics also differed significantly from the compounds resulting from the other two treatments. Focusing on 16 differential volatile metabolites, we further investigated their effects on flavor and quality, thus laying the foundation for a greater understanding of the biomarkers associated with changes in rice quality. This study contributes to a better understanding of the mechanisms underlying changes in rice quality after reduced nitrogen fertilizer application.

## 1. Introduction

Rice (*Oryza sativa* L.) is a herbaceous rice of the Poaceae family and is also the most important and ancient food in the rice genus [1]. It is rich in micronutrients, has strong planting adaptability and has a stable and high yield, giving it high economic value [2]. The northeast is the largest *japonica* rice-producing area in China, with a planting area of approximately 50% of the total *japonica* rice area nationwide [3]. Among them, SuiJing 18, which has a strong rice aroma and a soft glutinous texture, is particularly favored by consumers; its planting area in China is large, and its quality is reliably high [4,5]. For these reasons, we adopted SuiJing 18 as the material subject of our investigation of the effect of reduced nitrogen fertilizer.

Nitrogen is one of the most important nutrients required for rice growth and has a critical impact on rice yield and quality [6]. Increased fertilizer nitrogen inputs, especially the increased application of nitrogen fertilizer, have greatly contributed to increased global crop yields [7]. However, in further researching and developing green agriculture, it has emerged that excessive nitrogen fertilizer application can lead to a significant reduction in its efficiency, as well as decreased fertility and damage to the ecological environment [8,9].

Nitrogen fertilizer application may be one of the main factors influencing the aromatic composition of rice. Consumers have expressed strong interest in the quality of rice in recent years [10]. In addition, with the globalization, migration, and diversification of food choices, rice consumption is increasing in both local areas of production and non-rice-producing regions [11]. The main characteristics dictating the quality of rice are its milling, appearance, cooking, eating, and nutritional properties. Among these, rice’s eating quality is the most important characteristic, as it determines the price of rice in the market and influences customer satisfaction [12]. Taste and aroma are some of the most important factors of high-quality rice and determine whether rice is accepted by the public or not [13,14]. Rice with a better aroma and flavor is more likely to be accepted by consumers and will command a higher price in the market. Therefore, the evaluation and research of rice flavor play an important role in promoting both competition in the rice market and agricultural economic development.

Existing research on rice aroma has largely focused on preliminary analyses of the volatile compounds in rice or the characterization of its flavor quality using chromatography. In terms of objective evaluation, in addition to flavor analyzers and electronic noses, gas chromatography (GC) combined with corresponding pretreatment techniques and detectors with different characteristics has been widely explored and applied. With the invention, development, and application of gas chromatography, more specifically the establishment of GC–mass spectrometer (GC-MS) technology, research on rice flavor has made great progress [15,16,17]. Researchers [18,19] have analyzed the flavors in rice using GC-MS, established systems for evaluating rice flavor, and identified more than 500 volatile compounds in rice via GC-MS and other chromatographic techniques [13]. Volatile aromatic compounds in rice include aldehydes, ketones, organic acids, alcohols, esters, hydrocarbons, phenols, pyrazines, and pyridines [14,20]. A select few volatile organic compounds give rice its overall aroma and play a major role in sensory characterization; such compounds are referred to as key aroma components. Among them, 2-acetyl-1-pyrroline (2-AP) was first reported in 1982 [21] and is considered a typical aromatic compound of rice due to its extremely low sensory threshold, strong popcorn aroma, and sweetness [22]. It has been detected only in aromatic rice and is a typical substance therein [23,24]. That said, alternative key aroma components may be present at levels below the flavor threshold, and these compounds may interact with aromatic compounds to affect the overall flavor. In addition, aromatic compounds often present different flavor properties at different concentrations and may play a variety of roles in various aroma systems. Any rice aroma is influenced by the comprehensive performance of volatile organic compounds (VOCs) in different concentrations and interactions between compounds [25].

Nitrogen is critical for rice yield and quality, but its overuse can be detrimental to efficiency and the environment. In this study, we investigated the effects of reduced nitrogen on the quality of the northern *japonica* rice variety SuiJing 18, quantified the aromatic substances in rice by means of volatile metabolomics, and identified the key odor-active compounds that are essential to the aroma of SuiJing 18. In doing so, we have produced important references for the future application of agricultural and nitrogen fertilizers.

## 2. Materials and Methods

### 2.1. Materials and Reagents

SuiJing 18 was collected in Suihua, in the Heilongjiang province of northern China, in October 2023. The main stem of this variety has 12 leaves; it is of the long grain type and has a plant height of about 104.0 cm and a spike length of about 18.1 cm. There are around 108.8 grains per spike, with 1000 grains weighing about 26.0 g. Over two years, a quality analysis returned the following results. Brown rate: 80.9~82.2%; refined rate: 67.2~72.3%; chalky white grain rate: 4.0~10.0%; roughness: 80.9~82.2%; whole refined rice rate: 67.2%~72.3%; chalky white rice rate: 4.0~10.0%; chalkiness: 0.8~2.6%; straight-chain amylose content (dry basis): 17.67~19.11%; and gelatin consistency: 70.0 mm~73.0 mm. A three-year inoculation resistance appraisal returned the following results: leaf blight: 1~3; spike and neck blight: 1. A three-year cold resistance appraisal returned a treatment empty shell rate of 4.94~8.59%. Three nitrogen fertilizer reduction groups (conventional fertilization; 20% reduction in nitrogen fertilizer; and 40% reduction in nitrogen fertilizer) were created, as shown in Table 1. The nitrogen fertilizer used in this study was a solid granular fertilizer, the main components of which were urea (Yuntianhua, KunMing, Yunnan, China), diammonium (Yuntianhua, KunMing, Yunnan, China), and potassium sulfate (Yuntianhua, KunMing, Yunnan, China). We applied 85 kg of pure nitrogen per hectare, with a nitrogen–phosphorus–potassium ratio of 2:1:1. All the phosphorus fertilizers were used as basal fertilizers, and the potash fertilizers were applied twice as basal fertilizers and spike fertilizers, with 22.5 kg and 20 kg applied each time. The base fertilizer–tiller fertilizer–spike fertilizer–grain fertilizer ratio was 4:3:2:1, for which we used 34 kg of pure nitrogen, 42.5 kg of pure phosphorus, and 22.5 kg of pure potassium for the base fertilizer. We used 25.5 kg of pure nitrogen for the tiller fertilizer; 17 kg of nitrogen and 20 kg of pure potassium for the spike fertilizer; and 8.5 kg of pure nitrogen in the grain fertilizer. These levels were chosen based on previous studies that have demonstrated the significant effect of moderate fertilizer reductions on crop quality. The middle layer’s clayey meadow soil was mainly composed of black soil with a blocky structure. The soil pH was 6.6. The organic carbon and total nitrogen contents of the soil were 11.48~36.39 g/kg and 1.12~4.62 g/kg, respectively, across the entire year. The weather conditions in Suihua during the SuiJing 18 sowing and harvesting periods are exhibited in Table 2. Three replications were made, with 18 plots of 50 m^2^ each. The routine fertilization, 20% reduction in nitrogen fertilizer, and 40% reduction in nitrogen fertilizer are hereafter referred to as A1, A2, and A3.

NaCl was obtained from Sinopharm (Beijing, China). GC and HPLC-grade hexane was purchased from Merck (Darmstadt, Germany). The standard product was purchased from Sigma-Aldrich (Shanghai, China), and 3-hexanone-2,2,4,4-d4 was purchased from CDN (Quebec, QC, Canada).

### 2.2. Eating Quality

Eating quality tests were performed according to Zhang et al. [26], with a slight modification. The metrics for evaluating the eating quality evaluation of rice are presented in Table 3. Each sample was evaluated by nine panelists (five women and four men aged from 22 to 60) who were well trained in the principles and concepts of sensory evaluation, each having more than 3 years of experience.

### 2.3. Sample Preparation

For the analysis of eating quality, 1 kg of rice was divided by a sample splitter (SPD 4200, PerkinElmer, Waltham, MA, USA) prior to evaluation. For the analysis of GC-MS, three points were selected in each planting area. Three rice plants were cultivated at each point. The collected rice materials were immediately frozen in liquid nitrogen and stored at −80 °C before analysis. All rice samples collected in each planting area were ground to a powder in liquid nitrogen. Then, 500 mg was taken out for GC-MS analysis. A total of 9 samples were tested. The samples were ground to a powder in liquid nitrogen. Then, 500 mg (1 mL) of the powder was transferred immediately to a 20 mL head-space vial (Agilent, Palo Alto, CA, USA) containing a NaCl saturated solution in order to inhibit any enzyme reaction. The vials were sealed using crimped-top caps with TFE–silicone headspace septa (Agilent, Palo Alto, CA, USA). At the time of the solid-phase microextraction (SPME) analysis, each vial was placed in 60 °C for 5 min, following which a 120 µm DVB/CWR/PDMS fiber (Agilent, Palo Alto, CA, USA) was exposed to the headspace of the sample for 15 min at 60 °C. A temperature of 60 °C was chosen based on previous studies that demonstrated the highest efficiency in the extraction of VOCs.

### 2.4. GC-MS Conditions

After sampling, the desorption of the VOCs from the fiber coating was carried out in the injection port of the GC apparatus (8890; Agilent, Palo Alto, CA, USA) at 250 °C for 5 min in the splitless mode. The identification and quantification of VOCs were carried out using an Agilent 8890 and a 7000D MS (Agilent, Palo Alto, CA, USA) equipped with a 30 m × 0.25 mm × 0.25 μm DB-5MS (5% phenyl-polymethylsiloxane) capillary column. Helium was used as the carrier gas at a linear velocity of 1.2 mL/min. The injector temperature was kept at 250 °C. The oven temperature was programmed from 40 °C (3.5 min), increasing by 10 °C/min to 100 °C, by 7 °C/min to 180 °C, and by 25 °C/min to 280 °C, and held for 5 min. Mass spectra were recorded in electron impact (EI) ionization mode at 70 eV. The quadrupole mass detector, ion source, and transfer line temperatures were set at 150, 230, and 280 °C, respectively. The selected ion monitoring (SIM) mode was used for the identification and quantification of analytes.

### 2.5. Data Processing

Statistical analysis was performed using Origin Pro (OriginLab, Northampton, MA, USA) and SPSS 22.0 (IBM, Armonk, NY, USA). OPLS-DA was analyzed using SIMCA 14.1 (Umetrics, Umeå, Sweden). Heatmaps were created and a cluster analysis performed using the OmicStudio tool: https://www.omicstudio.cn (accessed on 30 May 2024). The differential analysis was completed by variance. *p* < 0.05 indicated a statistically significant difference, while *p* < 0.01 represented an extremely significant difference. Differential volatile metabolites were selected on the basis of VIP values >1 in the OPLS-DA analysis and *p* < 0.05.

## 3. Results and Discussion

### 3.1. Effect of Nitrogen Fertilizer Reduction on Eating Quality of SuiJing 18

**Figure 1:**
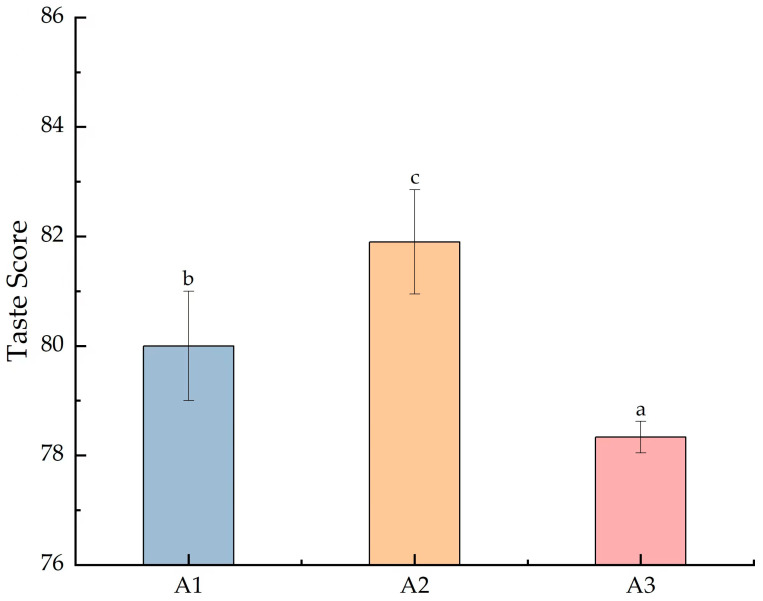
Effect of reduced nitrogen fertilizer on the eating quality of SuiJing 18. (α = 0.05; different letters indicate significant differences between groups).

In Figure 1, the *y*-axis represents the composite eating quality score calculated based on the sensory parameters of the rice. After the nitrogen fertilizer reduction, the eating quality of SuiJing 18 differed significantly, with a composite score of 80; when the nitrogen fertilizer was reduced to 40%, the composite score of the eating quality decreased significantly to 78.3. Meanwhile, when the 20% treatment with conventional irrigation and weight loss was applied, the composite score of the eating quality of SuiJing 18 reached a peak, at 81.9. The results of the sub-items showed that the odor and taste indicators of the samples of the treatment groups were higher than those of the remaining two groups; observing these results, we hypothesized that the contents of volatile metabolites, etc., were significantly different from those of the other treatments under this condition.

### 3.2. Effect of Nitrogen Fertilizer Reduction on Volatile Metabolomics of SuiJing 18

#### 3.2.1. Qualitative and Quantitative Volatile Metabolomics Analysis

Within GC-MS analysis, the matrix effect should be considered. Generally, in this study, matrix effects (MEs) were calculated by slope differences using the following equation: ME (%) = 100% × (slope of calibration curve in matrix/slope of calibration curve in solvent −1). MEs were divided into “no MEs” (−20 < no MEs < 20%), “medium MEs” (20–50%), and “strong MEs” (MEs > ±50%) [27,28]. The internal standard used in this study was 3-hexanone-2,2,4,4-d4, which has a slope of 1085.7 in the solution and 1096.7 in the matrix. The ME calculated according to the above equation was 0.6, which reveals that the matrix effect can be neglected in the present method.

**Figure 2:**
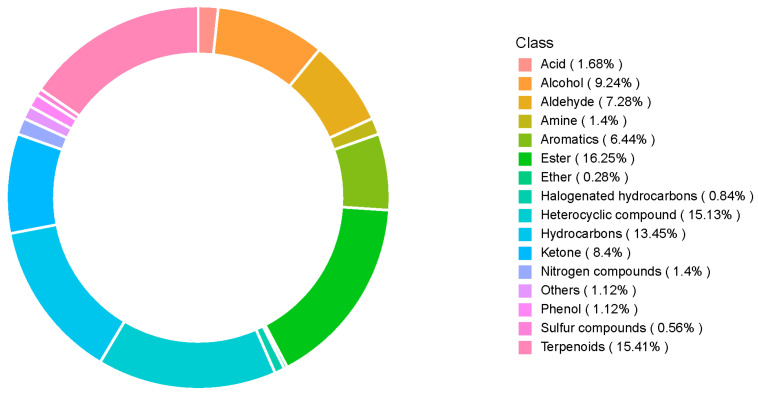
Effect of nitrogen fertilizer reduction on the volatile metabolite category composition of SuiJing 18.

We then compared the volatile compound contents of SuiJing 18 after different treatments with reduced nitrogen fertilization, as measured by GC-MS. Figure S1 shows the total ion chromatogram of the mixed samples. Table S1 lists all the VOCs detected in SuiJing 18. After the removal of compounds that contributed less to the aroma and the common column loss impurities, 15 classes of aromatic compounds were identified, totaling 358 aromatic components, among which esters, terpenoids, heterocyclic compounds, and hydrocarbons accounted for higher percentages (16.25%, 15.41%, 15.13%, and 13.45%, respectively) (Figure 2). We hypothesized that some of the volatile compounds may be closely related to the aromatic components of *japonica* rice in different treatments.

#### 3.2.2. Volatile Metabolite rOAV Analysis

**Figure 3:**
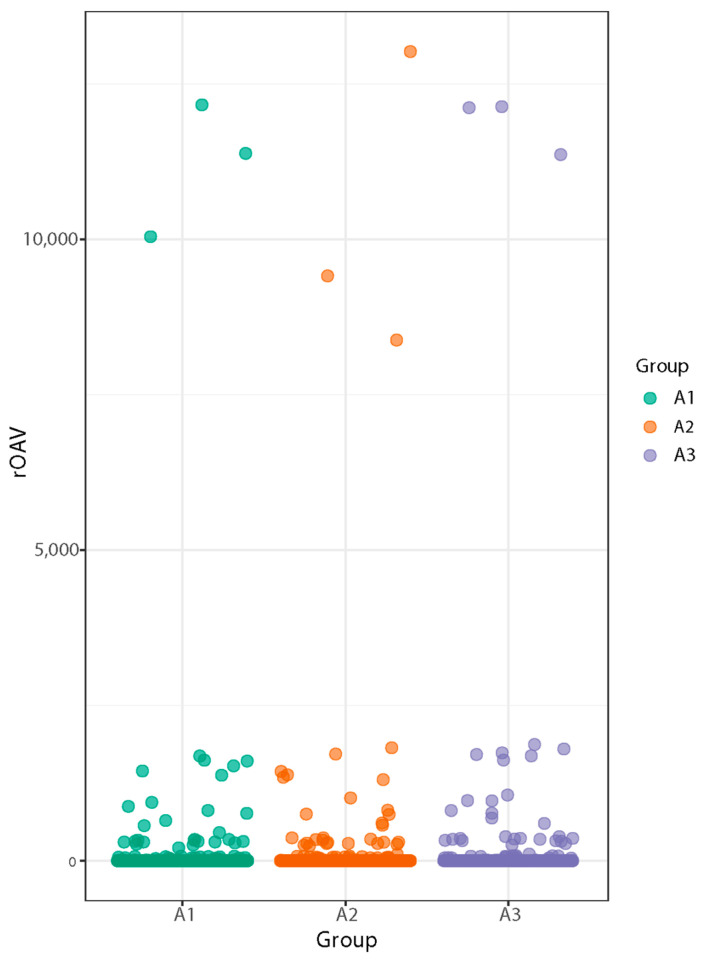
Effect of nitrogen fertilizer reduction on the volatile metabolite rOAV odor activity values of SuiJing 18.

The rOAV (relative odor activity value) represents the importance of each volatile compound’s contribution to the overall aroma. It is calculated as the ratio of the concentration of the compound to the olfactory detection threshold. Figure 3 shows that the compounds contributing to the aroma of SuiJing 18 were (Z,Z)-3,6-nonadienal, 2-thiophenemethanethiol, and (Z)-6-nonenal, with 2-methyltetrahydrofuran-3-one making the greatest contribution [29,30].

#### 3.2.3. OPLS-DA Analysis

**Figure 4:**
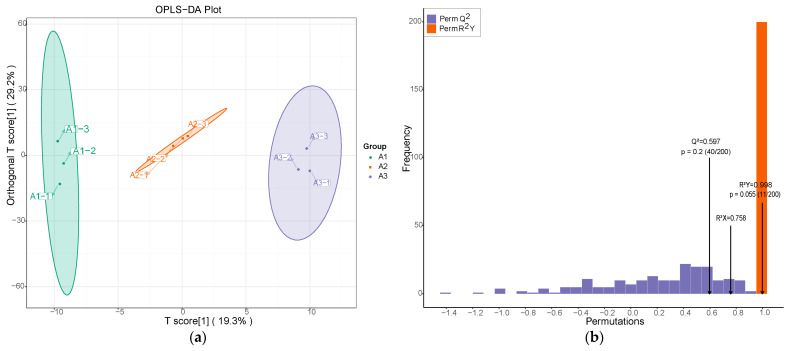
Effect of reduced nitrogen fertilizer on the metabolic variation of SuiJing 18: (**a**) OPLS-DA score plot; (**b**) permutation testing of OPLS-DA in A1 vs. A2 vs. A3 group.

We used OPLS-DA analysis to compare the aromatic components of SuiJing 18 from three different nitrogen fertilizer treatments [31]. As demonstrated in Figure 4a, the vertical axis represents the predicted principal components, and the horizontal axis represents the orthogonal principal components. The principal components were predicted to make up 19.3% of the different nitrogen fertilizer treatment groups, which indicated that the metabolites of routinely applied nitrogen fertilizers differed significantly from those resulting from reduced nitrogen fertilizer application. The orthogonal principal components made up approximately 29.2% of A1, A2, and A3. According to the OPLS-DA analysis, volatile metabolomics is able to completely differentiate the three groups of different nitrogen fertilizer treatments. This indicates that the volatile compounds underwent significant changes when the amount of applied nitrogen fertilizer was reduced. To further confirm the model, a permutation test was performed. The high values of R^2^X, R^2^Y, and Q^2^ in Figure 4b indicate the reliability of the OPLS-DA model in classifying different nitrogen fertilizer treatments [32].

#### 3.2.4. Differential Volatile Metabolite Screening

**Figure 5:**
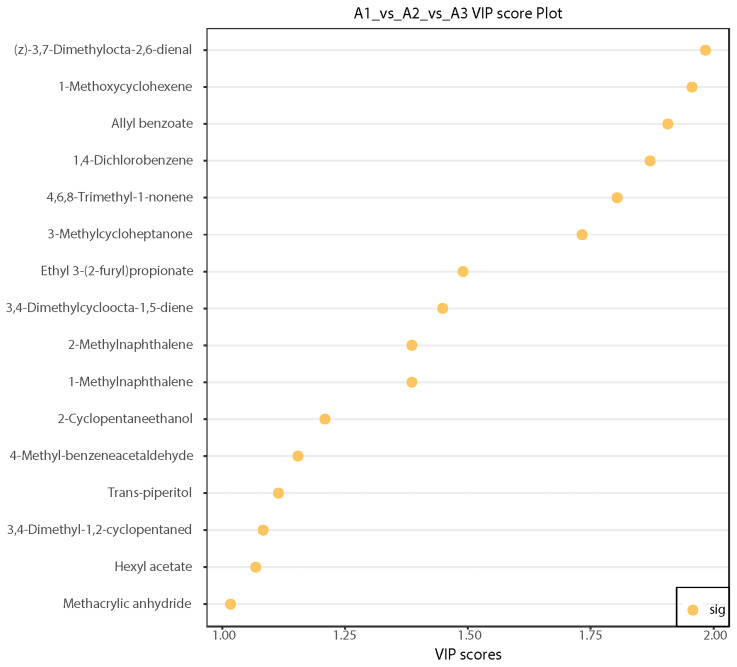
Plot of VIP values of differential volatile metabolites from different nitrogen fertilizer treatment groups.

The reduced application of nitrogen fertilizer induced changes in 16 volatile metabolites in SuiJing 18, including 3,4-dimethylcycloocta-1,5-diene, 4,6,8-trimethyl-1-nonene, (z)-3,7-dimethylocta-2,6-dienal, 3,4-dimethyl-1,2-cyclopentaned, 2-cyclopentaneethanol, ethyl 3-(2-furyl)propionate, hexyl acetate, and allyl benzoate (Table 4). The 16 key compounds were selected on the basis of VIP values >1 derived from the OPLS-DA analysis, which indicated that these compounds contribute significantly to differentiation between treatments. The differences in the contents of the 16 aroma components with VIP > 1 in A1, A2, and A3 were examined in the different treatments of SuiJing 18. Significant differences were found in the contents of (z)-3,7-dimethylocta-2,6-dienal, 1-methoxycyclohexene, allyl benzoate, and other compounds in the different treatments (Figure 5). Among them, (z)-3,7-dimethylocta-2,6-dienal had a sweet and distinct lemon aroma, 1-methoxycyclohexene had a grassy and spicy aroma, and allyl benzoate had sweet, floral, and cherry flavors. Nitrogen reduction may alter sugar and ester metabolism, leading to increased sweetness and floral aromas [33]. Therefore, with a decrease in the amount of nitrogen fertilizer applied, the faint sweet flavor of SuiJing 18 became more prominent.

#### 3.2.5. Analysis of Key Differential Volatile Metabolites

**Figure 6:**
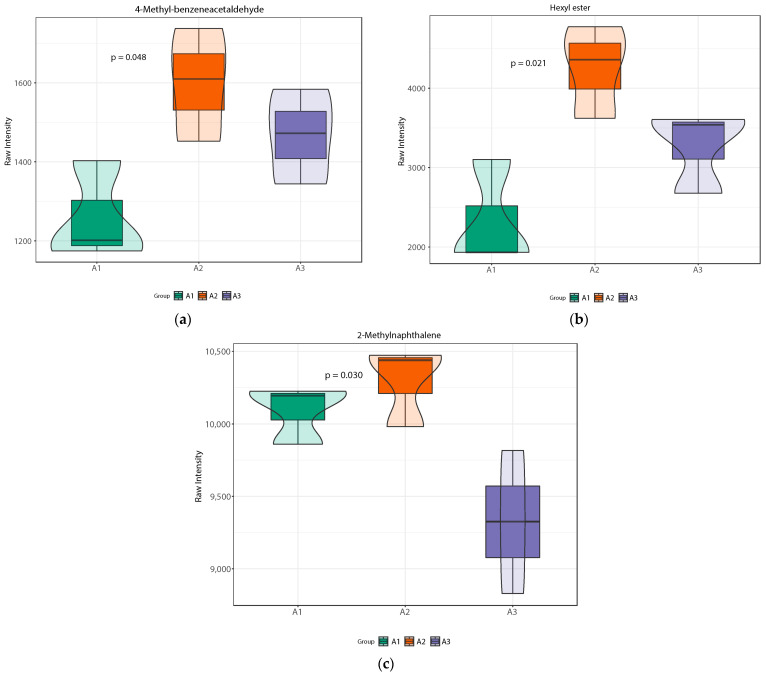
Violin plots of differential volatile metabolites within nitrogen fertilizer treatment groups: (**a**) 4-methyl-benzeneacetaldehyde; (**b**) hexyl acetate; (**c**) 2-methylnaphthalene.

Figure 6 shows a violin plot of the differences in the relative content of volatile metabolites between each group. The different metabolites are roughly divided into two groups in Figure 6, which are dictated by the trends in the application of nitrogen fertilizer. The first trend involves a gradual reduction in nitrogen fertilizer after an initial increase, while the other involves only a decrease. The relative content of the key sweet compounds 4-methyl-benzeneacetaldehyde, hexyl acetate, and 2-methylnaphthalene first increased and then decreased; the sweetness of the resulting rice was highest when nitrogen fertilizer was reduced by 20%. At the same time, the influence of 4-methyl-benzeneacetaldehyde on flavor cannot be ignored, as this compound has a distinct green grass flavor. The compound 4-methyl-phenylacetaldehyde also has a green-grass-like flavor. Hexyl acetate has a significant impact on fruit aroma, while 2-methylnaphthalene has a significant effect on floral aroma. Through an analysis of multiple differential volatile metabolites, we inferred that the flavor of SuiJing 18 would be improved with a reduction in nitrogen fertilization, which was also consistent with the results of the eating quality evaluations.

#### 3.2.6. Hierarchical Cluster Analysis of Differential Volatile Metabolites

**Figure 7:**
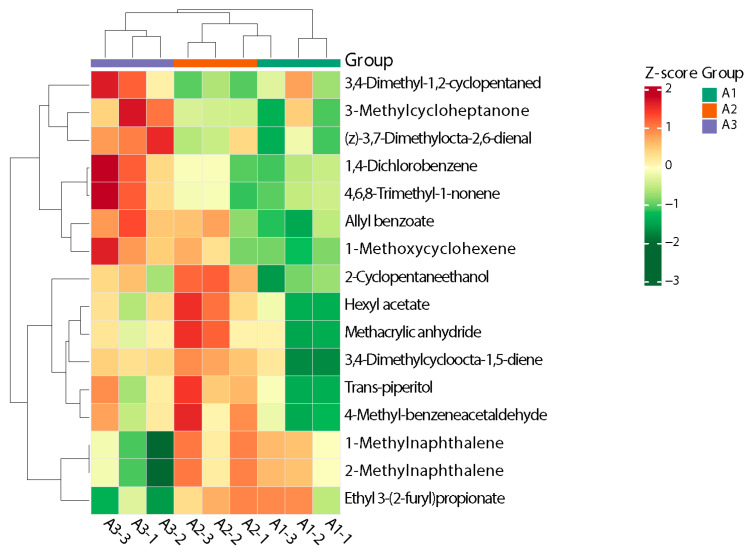
Heatmap of clustering of differential volatile metabolites in different nitrogen fertilizer treatment groups.

The heatmap clearly exhibited the changes in the content of the 16 volatile metabolites in each group. Z-score standardization is the standardization of data based on the mean and standard deviation of the raw data. The different colors were filled with the different values obtained after normalization for different relative contents (red for high content, green for low content). As shown in Figure 7, the aromatic components were largely clustered in four categories [34]. Zone I was dominated by 3-methylcycloheptanone and (z)-3,7-dimethylocta-2,6-dienal, and these volatile metabolites were significantly higher in A3 than in A1 and A2. The lowest content was found in A1, which basically showed a decreasing trend with an increase in the amount of nitrogen fertilizer applied. Zone II was dominated by 1,4-dichlorobenzene, 4,6,8-trimethyl-1-nonene, and 1-methylnaphthalene, which showed basically the same trend as zone I and could be clearly distinguished from A3. The compounds detected in zone III were 2-methyl-2-propenoic acid anhy, 3,4-dimethylcycloocta-1,5-diene, and trans-piperitol, which were found at low levels in most of the samples of A1 and detected in all other samples. The compounds present in zone IV were 2-methylnaphthalene, 1-methylnaphthalene, 2-furanpropanoic acid, and ethyl ester, which did not differ significantly; the only exception to this rule were the low levels detected in a portion of A3, which suggests that when nitrogen fertilizer is reduced by 40% or more, the levels of these compounds decrease significantly.

Overall, A3 could be distinguished from A1 and A2 more clearly, and A1 and A2 could be distinguished from a few differential metabolites, although they did not differ much in their content of some metabolites; this observation is consistent with the results of the OPLS-DA model described above.

#### 3.2.7. Correlation Analysis of Differential Volatile Metabolites

**Figure 8:**
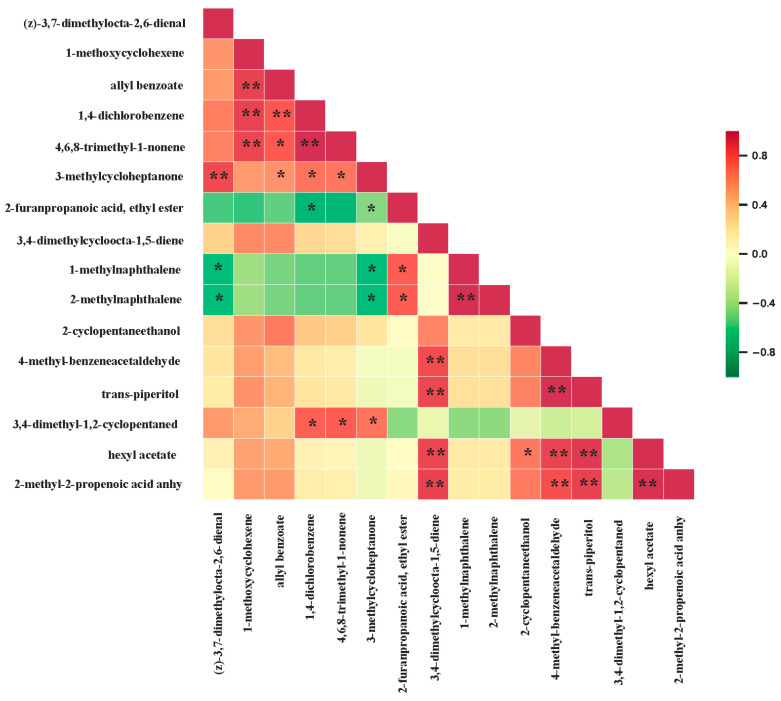
Pearson correlation plot of differential volatile metabolites in various nitrogen fertilizer treatment groups (** means *p* < 0.01; * means 0.01 < *p* < 0.05).

In order to identify the synergy or mutual exclusivity in the relationships between different metabolites, we performed a correlation analysis of the differential metabolites in A1, A2, and A3 to provide further insight into these inter-regulatory relationships (Figure 8). The results of this analysis showed a significant positive correlation between 3-methylcycloheptanone and (z)-3,7-dimethylocta-2,6-dienal, which is consistent with the results obtained above. A3 can be clearly distinguished from A1 and A2 by these two metabolites, as their contents were the highest in A3 and gradually decreased with the increase in the amount of nitrogen applied. Furthermore, 1-methylnaphthalene and 2-methylnaphthalene showed a significant negative correlation with (z)-3,7-dimethylocta-2,6-dienal. The contents of 1-methylnaphthalene and 2-methylnaphthalene were smaller in A3, while their contents in A1 and A2 were significantly greater than that in A3 (Table 4), further confirming that we could distinguish A3 by these two metabolites. These analyses therefore reiterated that we could recognize the amount of nitrogen applied to SuiJing 18 by the contents of these compounds.

#### 3.2.8. KEGG Analysis of Differential Volatile Metabolites

**Figure 9:**
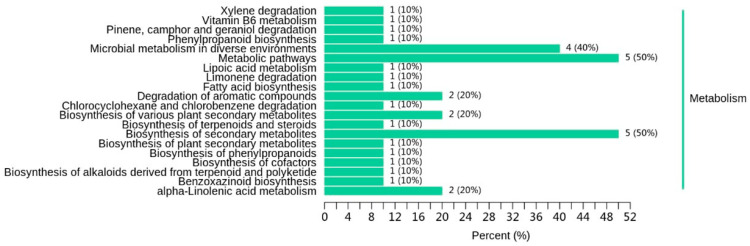
Effect of reduced nitrogen fertilizer on the differential metabolite pathway of SuiJing 18 (A3 compared with A1).

Individual metabolites interact and form distinct pathways. We focused on the metabolic pathways of differential volatile metabolites. Table 4 lists the differential volatile metabolites among A1, A2, and A3. The KEGG pathways involved in the differential volatile metabolites between A1 and A3 were the microbial metabolism in diverse environments; the degradation of aromatic compounds; the biosynthesis of various plants’ secondary metabolites; and alpha-linolenic acid metabolism, among others (Figure 9). Metabolomics analysis is generally used for the characterization of rice’s aging process and traceability to provide a potential pathway and identify any changes in rice quality [32,35,36]. In accordance with the present study, both Liu et al. [35] and Wang et al. [32] have found that the alpha-linolenic acid metabolism is related to a deterioration in rice quality.

#### 3.2.9. Flavoromics Analysis of Differential Volatile Metabolites

**Figure 10:**
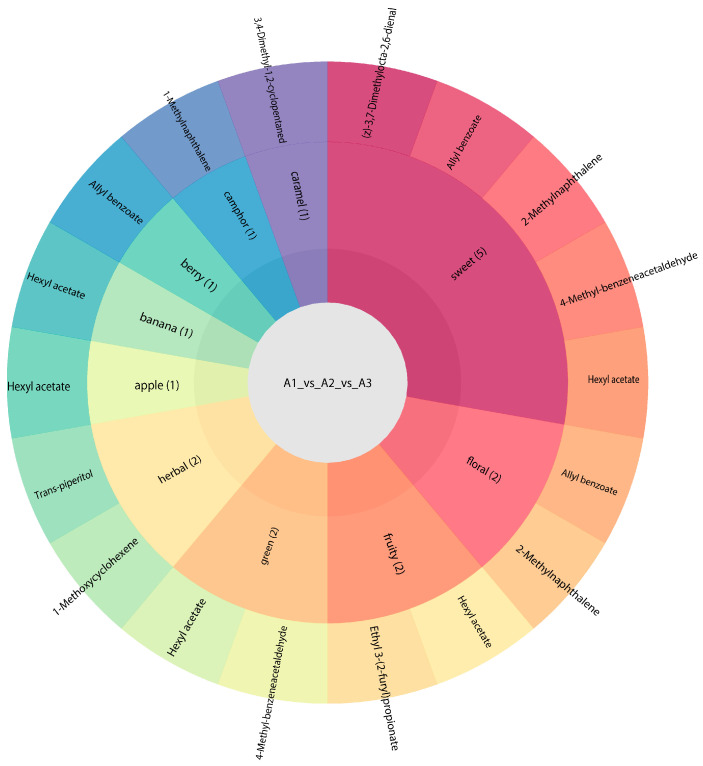
Effect of reduced nitrogen fertilizer on the differential metabolite flavor of SuiJing 18.

**Figure 11:**
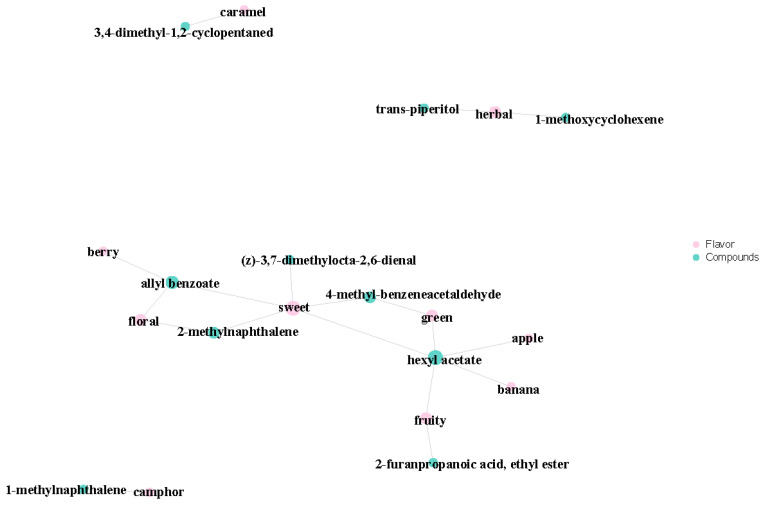
Plot of the sensory flavor profile and differential metabolite association network of different nitrogen fertilizer treatment groups.

We carried out a sensory analysis of differential volatile metabolites to examine the sensory flavor profiles in the samples. We focused on annotating the 10 most dominant sensory flavor features with differential volatile metabolites before identifying those with the highest number of annotations [37]. Our results indicated that the differential volatile metabolites of A1, A2, and A3 largely affected the sweetness of the rice’s overall flavor (Figure 10). Various differential volatile metabolites were proven to provide sweetness, including (z)-3,7-dimethylocta-2,6-dienal, allyl benzoate, 2-methylnaphthalene, 4-methyl-benzeneacetaldehyde, and hexyl acetate. We found sweetness to be the most important flavor characteristic of SuiJing 18; the compounds allyl benzoate and 4-methyl-benzeneacetaldehyde have the most significant effect on this quality. The other most influential aromas (in ascending order) on the flavor of SuiJing 18 are the grassy aroma, floral aroma, and fruity aroma. The role of 2-methylnaphthalene in the floral aroma cannot be ignored. Grassy aroma also has a significant influence on the flavor of SuiJing 18 and is largely provided by trans-piperitol and 1-methoxycyclohexene. The other flavor profiles of SuiJing 18 include caramel, camphor, berry, banana, and apple flavors. As shown in Figure 11, we found that hexyl acetate had a significant effect not only on sweetness but also on the apple, banana, and grassy aromas. Allyl benzoate significantly contributed to sweetness, the berry flavor, and floral aroma. Therefore, we concluded that as the amount of nitrogen fertilizer applied was reduced, the levels of allyl benzoate and 4-methyl-phenylacetaldehyde in SuiJing 18 gradually increased, and the sweetness of the rice also increased. This may be related to the increase in the starch content of SuiJing 18, which was caused by the reduction in the nitrogen fertilizer applied.

The volatile compounds released from the rice depended not only on the amounts of these molecules in rice, but also on their interaction with other ingredients, which then affected the capability of the release. The effect of nitrogen reduction on other components should be considered in this study, especially as it pertains to proteins. As complex systems with the presence of several components (such as hydrocolloids and active compounds), the aggregation of proteins will always vary between foods. Zhu et al. [38] concluded that two pathways largely affect the aggregation of proteins. One is that of protein conformation, which is affected by factors such as temperature, mechanical forces, pressure, etc. [39,40]. The other is that of driving forces, such as pH, protein source, etc., and their interactions [41]. In the future, our team will conduct in-depth research on the impact of protein changes caused by reduced nitrogen fertilizer on the quality of SuiJing 18.

## 4. Conclusions

The effects of nitrogen fertilizer reduction on the volatile metabolomics of SuiJing 18 were investigated in this study. The results showed that a reduction in the application of nitrogen fertilizer could improve the eating quality of rice. The volatile metabolomics detected 358 volatile compounds in SuiJing 18 plants from different nitrogen fertilizer treatment groups, including 16.25% esters, 15.41% terpenoids, 15.13% heterocyclic compounds, and 13.45% hydrocarbons. The types and contents of volatile compounds differed between the three treatments. There were 16 differential metabolites in SuiJing 18 after the application of nitrogen fertilizer was reduced; sweetness was found to be the most important flavor characteristic of rice. The key compounds affecting the sweetness of SuiJing 18 were (z)-3,7-dimethylocta-2,6-dienal, allyl benzoate, 2-methylnaphthalene, hexyl acetate, and 4-methyl-benzeneacetaldehyde, among which allyl benzoate and 4-methyl-phenylacetaldehyde had the most significant effect on sweetness. The results of our cluster analysis showed that the key sweet compounds 2-methylnaphthalene, 4-methyl-benzeneacetaldehyde, and hexyl acetate showed a trend of first increasing and then decreasing with the reduction in nitrogen fertilizer. The sweetness of rice also reached a peak when the nitrogen fertilizer was reduced by 20% (68 kg of pure nitrogen per hectare), which may be related to an increase in the starch content of Suijin 18 within a certain range of nitrogen fertilizer reduction and also reinforced the results of our eating quality evaluations. In conclusion, the sweet flavor of SuiJing 18 was most obvious after a 20% reduction in nitrogen fertilizer (68 kg of pure nitrogen per hectare) relative to conventional fertilization (85 kg of pure nitrogen per hectare). Its sweetness was enhanced and its eating quality improved by this process. Therefore, through a reduction in nitrogen fertilizer, not only can the rate of nitrogen fertilizer utilization be improved, the aromatic quality of *japonica* rice can also be enhanced. The results of this study may be used to optimize the use of nitrogen fertilizer in different rice-growing regions, serving to reduce its environmental impact and improve the quality of produce.

## Figures and Tables

**Table 1:** Nitrogen fertilizer reduction patterns.

Scale	5 m	5 m	5 m
10 m	A2 20% reduction in nitrogen fertilizer	A1 conventional fertilization	A3 40% reduction in nitrogen fertilizer
10 m	A3 40% reduction in nitrogen fertilizer	A2 20% reduction in nitrogen fertilizer	A1 conventional fertilization
10 m	A1 conventional fertilization	A3 40% reduction in nitrogen fertilizer	A2 20% reduction in nitrogen fertilizer

**Table 2:** Weather conditions in Suihua during the SuiJing 18 sowing and harvesting periods.

Month		April	May	June	July	August	September
Ten Days of a Month							
Item							
Average temperature (°C)	First	6.5	13.0	17.0	23.0	21.4	18.7
	Middle	5.6	10.8	21.3	22.2	23.7	15.9
	End	6.9	17.3	22.8	24.4	20.4	14.2
Amount of precipitation (mm)	First	0.0	0.1	11.1	76.3	26.4	1.0
	Middle	9.8	23.9	8.4	63.4	21.4	19.5
	End	18.2	18.3	80.8	15.0	36.5	34.7
Sunshine time (h)	First	56.1	84.1	64.6	88.9	65.6	70.7
	Middle	72.4	69.5	90.1	68.8	59.3	68.4
	End	74.2	81.7	48.7	52.9	22.9	43.2

**Table 3:** Metrics for evaluating the eating quality of rice.

Index	Score
Odor	0~20
Color	0~7
Glossiness	0~8
Integrity	0~7
Stickiness	0~10
Elasticity	0~10
Hardness	0~10
Taste	0~25
Texture of cold rice	0~5
Composite eating quality score	0~100

**Table 4:** Differences in volatile metabolites between the three treatments.

Class	Index	Compounds	VIP	*p*-Value	FC		
					A2 vs. A1	A3 vs. A1	A3 vs. A2
Hydrocarbons	XMW0771	3,4-Dimethylcycloocta-1,5-diene	1.45	0.032	2.78	2.42	0.87
Hydrocarbons	XMW0121	4,6,8-Trimethyl-1-nonene	1.80	0.018	1.03	1.24	1.20
Terpenoids	KMW0432	(z)-3,7-Dimethylocta-2,6-dienal	1.98	0.008	1.14	1.43	1.26
Terpenoids	KMW0420	Trans-piperitol	1.11	0.049	1.39	1.23	0.89
Ketone	KMW0260	3,4-Dimethyl-1,2-cyclopentaned	1.08	0.034	0.97	1.04	1.08
Alcohol	WMW0103	2-Cyclopentaneethanol	1.21	0.006	1.41	1.23	0.87
Heterocyclic compound	D81	Ethyl 3-(2-furyl)propionate	1.49	0.034	1.02	0.87	0.85
Others	XMW1440	Methacrylic anhydride	1.02	0.044	1.48	1.24	0.84
Ester	KMW0204	Hexyl acetate	1.07	0.021	1.83	1.41	0.77
Aromatics	WMW0227	1,4-Dichlorobenzene	1.87	0.014	1.04	1.24	1.19
Aldehyde	D173	4-Methyl-benzeneacetaldehyde	1.15	0.048	1.27	1.16	0.92
Ester	XMW0776	Allyl benzoate	1.91	0.021	1.16	1.26	1.08
Ketone	XMW0039	3-Methylcycloheptanone	1.73	0.041	1.02	1.15	1.12
Aromatics	KMW0205	1-Methoxycyclohexene	1.96	0.020	1.13	1.26	1.11
Aromatics	w08×082	1-Methylnaphthalene	1.39	0.031	1.02	0.92	0.91
Aromatics	XMW0662×082	2-Methylnaphthalene	1.39	0.031	1.02	0.92	0.91

## Data Availability

Data are contained within the article and/or Supplementary Materials.

## Supplementary Materials

The following supporting information can be downloaded at: https://www.mdpi.com/article/10.3390/foods13203310/s1, Figure S1: The total ion chromatogram of mixed samples; Table S1: Complete list of measured volatile metabolite in SuiJing 18.
