# Supplementary material for: The Effect of Reduced Nitrogen Fertilizer Application on japonica Rice Based on Volatile Metabolomics Analysis
Source: Foods. 2024 Oct 18;13(20):3310. doi: 10.3390/foods13203310 (PMC11507305; doi:10.3390/foods13203310)
Supplement: Supplementary file 1 [file foods-13-03310-s001.zip › foods-3234650-Figure S1.pdf]

Study on the effect of nitrogen fertilizer reduction on SuiJing 18 based on volatile metabolomics analysis

Jiahao Wu [1,2], Qian Wang [2], Dong Zhang [2], Xiaoliang Duan[2] and Hui Sun [1,2,]

[1] University of Shanghai for Science and Technology, School of Health Science and Engineering, Shanghai 200093, PR China

[2] Academy of National Food and Strategic Reserves Administration, Beijing 100037, PR China;

*Corresponding author:

Dr. Hui Sun

Academy of National Food and Strategic Reserves Administration

No. 11, Baiwanzhuang street, Xicheng district, Beijing, P. R. China

Ph: 86-13601375755 E-mail: sh@ags.ac.cn

Supplementary Materials

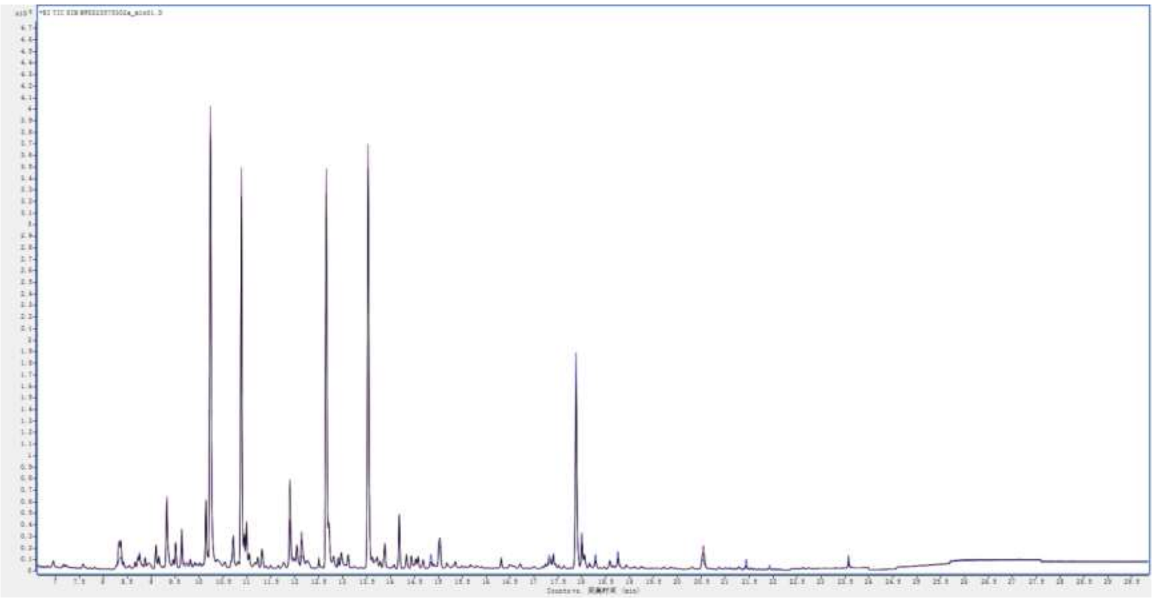

Figure S1. The total ion chromatogram of mixed samples.
